# Intramammary Immunization of Pregnant Mice with Staphylococcal Protein A Reduces the Post-Challenge Mammary Gland Bacterial Load but Not Pathology

**DOI:** 10.1371/journal.pone.0148383

**Published:** 2016-02-10

**Authors:** Jully Gogoi-Tiwari, Vincent Williams, Charlene Babra Waryah, Sangeetha Mathavan, Harish Kumar Tiwari, Paul Costantino, Trilochan Mukkur

**Affiliations:** 1 Curtin Health Innovation Research Institute, Curtin University, Bentley, Perth, Western Australia, 6102, Australia; 2 College of Veterinary Sciences and Animal Husbandry, Central Agricultural University, Selesih, Aizawl, Mizoram, 796014, India; 3 Department of Medicine and Cell Biology, Albert Einstein College of Medicine, 1300 Morris Park Ave, Bronx, NY, 10461, United States of America; 4 School of Veterinary and Life Sciences, Murdoch University, Murdoch, Perth, Western Australia, 6150, Australia; Midwestern University, UNITED STATES

## Abstract

Protein A, encoded by the *spa* gene, is one of the major immune evading MSCRAMM of *S*. *aureus*, demonstrated to be prevalent in a significant percentage of clinical bovine mastitis isolates in Australia. Given its’ reported significance in biofilm formation and the superior performance of *S*. *aureus* biofilm versus planktonic vaccine in the mouse mastitis model, it was of interest to determine the immunogenicity and protective potential of Protein A as a potential vaccine candidate against bovine mastitis using the mouse mastitis model. Pregnant Balb/c mice were immunised with Protein A emulsified in an alum-based adjuvant by subcutaneous (s/c) or intramammary (i/mam) routes. While humoral immune response of mice post-immunization were determined using indirect ELISA, cell-mediated immune response was assessed by estimation of interferon-gamma (IFN-γ) produced by protein A-stimulated splenocyte supernatants. Protective potential of Protein A against experimental mastitis was determined by challenge of immunized versus sham-vaccinated mice by i/mam route, based upon manifestation of clinical symptoms, total bacterial load and histopathological damage to mammary glands. Significantly (p<0.05) higher levels of IgG1 isotype were produced in mice immunized by the s/c route. In contrast, significantly higher levels of the antibody isotype IgG2a were produced in mice immunized by the i/mam route (p<0.05). There was significant reduction (p<0.05) in bacterial loads of the mammary glands of mice immunized by Protein A regardless of the route of immunization, with medium level of clinical symptoms observed up to day 3 post-challenge. However, Protein A vaccine failed to protect immunized mice post-challenge with biofilm producing encapsulated *S*. *aureus* via i/mam route, regardless of the route of immunization, as measured by the level of mammary tissue damage. It was concluded that, Protein A in its’ native state was apparently not a suitable candidate for inclusion in a cell-free vaccine formulation against mastitis.

## Introduction

Two most important immune evasion antigens of *Staphylococcus aureus* are Protein A and capsular polysaccharide [1–3]. Capsular polysaccharides (CP) are poorly immunogenic and have been evaluated as conjugate vaccines for their vaccine potential against *S*. *aureus* infections in humans [4,5]. However, neither Protein A in its’ native state nor as a conjugate vaccine has been tested for its’ potential as a vaccine candidate against mastitis. Protein A is one of the crucial membrane bound proteins expressed by *S*. *aureus* which has been reported to enhance its’ pathogenicity by suppressing immune system of the host [6] and resisting bacterial clearance from the host [1]. Of the two distinct terminal regions of spA, the C-terminal region binds to the cell wall peptidoglycan [7] whereas its’ N-terminal region contains highly conserved immunoglobulin binding domain D, which can bind to the Fcγ and Fab portions of IgG and IgM [7,8] via the variable region of the Fab heavy chain (VH) through framework residues, without the involvement of the hypervariable regions implicated in antigen recognition [9] leading to claimed suppression of both innate and adaptive immunity [10]. In addition, the immunoglobulin binding domains of SpA can further interact with von Willebrand factor (vWF) assisting in the adherence of *S*. *aureus* cells to vascular endothelial cells [11]. Protein A has been reported to inhibit opsonisation of *S*. *aureus* presumably due to steric hindrance to the complement-binding sites of immunoglobulins and preventing activation of the alternative complement pathway [12]. Further evidence for the importance of Protein A as a virulence factor was demonstrated by comparison of its’ pathogenicity using a *spa* knockout mutant strain using the murine bacteraemia model, when a significantly lower mortality of mice infected intravenously with the mutant strain of *S*. *aureus* versus the wild type parent strain was recorded [10,13].

Although Protein A is primarily claimed for its ability to suppress the humoral arm of immune response by inhibiting opsonophagocytosis [6,14], recent report of the potential role of Protein A in biofilm formation contributing to the severity of *S*. *aureus* associated infections, including implant related infections, endocarditis, cystic fibrosis in humans [15] and bovine mastitis [16,17] warrants attention. Protein A is one of the major microbial surface components recognizing adhesive matrix molecules (MSCRAMMs) encased in the biofilm matrix of *S*. *aureus* [18]. The importance of this molecule in biofilm formation was demonstrated using a murine subcutaneous catheter model in which the colony forming units recovered from the catheter contaminated with the *spa* deletion mutant were significantly less than those with the parent wild type [18].

The only available treatment for contagious mastitis due to *S*. *aureus* at present involves the use of antibiotics. However, due to development of antibiotic resistance by *S*. *aureus* [19,20] and ability of the pathogen to develop biofilm in mammary gland [17], a cure rate ranging from 0–52% from mastitis in lactating animals, which relapses in most of the cases [21], has been reported. A recent report demonstrating display of antimicrobial resistance even by methicillin sensitive *Staphylococcus aureus* (MSSA) isolates from bovine mastitis cases, when presented as biofilms, further complicates the treatment of bovine mastitis [22]. Given high cost of treatment with antibiotics, antibiotic residues in milk, emerging antibiotic resistance and severe mammary tissue damage, there is an urgent need to develop an effective vaccine against *S*. *aureus*-associated bovine mastitis [23]. Numerous efforts have been reported to develop an effective vaccine against bovine mastitis, but none have yet yielded satisfactory outcomes [24–30]. Several approaches for vaccine development including killed whole organisms [31], live attenuated vaccine [32], capsular polysaccharides [33] and poly- N-acetyl glucosamine (PNAG) [34]-based conjugate vaccines, DNA vaccine encoding the clumping factor [35] have been undertaken and claimed to reduce the clinical severity but not prevent intra-mammary infection (IMI).

Protein A can be a suitable candidate for prevention of *S*. *aureus*-associated infections due to its’ immune evasion property [36,37] because of the prevalence of *spa* gene in majority of clinical *S*. *aureus* isolates of human [38] and bovine origin [39], and demonstrated ability of modified SpA, devoid of antibody Fcγ or Fab binding capacity (spA_KKAA_), to protect against renal infection induced by intravenous challenge with methicillin resistant *S*. *aureus* (MRSA) [10,13,38] resulting in protection as judged by a reduction in bacterial load in renal tissue samples [10]. SpA_KKAA_ was also reported to boost humoral immune response by producing antibodies to neutralize Fcγ/Fab binding characteristics of SpA [13]. Furthermore, administration of mouse monoclonal antibody against modified SpA via intraperitoneal route was reported to protect neonatal mice against sepsis caused by *S*. *aureus* [40]. However, neither its’ immunogenicity nor protective potential against mouse mastitis, used as a model for bovine mastitis, has previously been reported. Given the presence of Protein A in the biofilm of *S aureus* [18], neutralization potential of SpA-specific monoclonal antibody [40], prevalence in majority of clinical *S*. *aureus* strains of bovine mastitis origin in Australia [39] and the superior immunogenicity and protective potential of *S*. *aureus* biofilm versus planktonic vaccine against bovine mastitis [16], it was of interest to determine the immunogenicity and protective potential of Protein A against bovine mastitis using the mouse mastitis model with a view to determining its’ suitability for inclusion in conjugate vaccine formulations, hence the aim of this study. While reports on the response of host(s) to immunization with Protein A and its’ potential protective role against various *S*. *aureus* associated systemic infections have been reported [10,13,36,38], no similar studies on the immunogenicity and protective potential of Protein A against *S*. *aureus*-associated mouse mastitis, used as a model for bovine mastitis in this study, have been reported. Data emerging from our investigation, presented in this communication, may provide important information enabling a decision on the suitability of Protein A in its native state as a potential carrier in the formulation of conjugate vaccines against *S*. *aureus* associated mastitis.

## Material and Methods

### Animal ethics approval

A total of 48 pregnant Balb/c mice (3^rd^ day) obtained from the Animal Resources Centre, Perth, Western Australia were used for the study. The animals were allowed to rest for 3 days upon arrival. All animal experiments were carried out with the approval of Curtin University’s Animal Ethics Committee (Approval No: AEC_2013_24) ensuring compliance with the Western Australian Animal Welfare Act, 2002. For the immunogenicity study, mice were euthanized on 28^th^ day of vaccination for collection of blood to obtain serum samples for antibody isotyping and collection of spleen for preparation of Protein A or Concanavalin A (ConA)-stimulated splenocyte supernatants for cytokine analysis, respectively. Following challenge with *S*. *aureus*, immunized and sham- immunized mice were observed for 5 days and euthanized on day 6 post-challenge. During this period mice were observed at 6 hourly intervals for the first two days and then twice daily in the remaining 3 days. The humane end points set for euthanasia of mice in the animal trials were the laboured movement, dehydration, body-weight loss greater than 10% and/or hunched back position of the animal indicative of moribund state.

### Immunization of mice

The experimental mice (n = 48), which were on 6^th^ day of pregnancy, were divided into four groups, each group comprising 12 mice (Table 1). Immunization of mice was carried out on day 0, 7, 14 and starting day was 6^th^ day of post-pregnancy. One group of mice was immunised with Protein A (Sigma Aldrich, PO Box 970 Castle Hill NSW 1765 Australia) by subcutaneous (s/c) route whereas the second group was vaccinated by intramammary (i/mam) route (Table 1). The remaining two groups (Groups 3 and 4) were sham-immunized with PBS by either s/c or i/mam routes, respectively (Table 1). Equal volumes of Protein A at 25μg/ml were emulsified with equal volume of Imject Alum (Thermo Fisher Scientific). A total 100 μl/dose was administered to the mice via s/c or i/mam routes, respectively. For i/mam inoculation, mice were anaesthetised with a dose of the anaesthetic comprising 100 mg ketamine kg−1 and 10 mg xylazine kg−1 administered by the intra-peritoneal (i/p) route, laid on their back under a binocular microscope, and the teats and the surrounding area were disinfected with 70% ethanol. The hind teats, numbered R5 and L5, were held with fine forceps and the duct orifice was located. A volume of 100 μl of protein A with adjuvant was inoculated through each teat orifice using blunt 31 gauge needles. Sham-vaccination of mice with PBS was carried out using the same protocol. Mice were monitored twice a day. Mice in the “immunogenicity experiment” group were euthanized on 28^th^ day for collection of blood and spleen samples for antibody isotyping and cytokine analysis, respectively.

**Table 1:** Experimental groups of mice used in the immunogenicity and protective potential trials of Protein A vaccines (n = 48).

Sl. No	Group	Number of mice used for determination of immunogenicity	Number of mice used for determination of protective potential
1	Protein A s/c route	6	6
2	Protein A i/mam route	6	6
3	PBS control for s/c route	6	6
4	PBS control for i/mam route	6	6

### Detection of antigen-specific antibody isotypes in serum

Briefly, Nunc-Immuno^™^ MicroWell^™^ 96 well solid plates (Sigma Aldrich Pty Ltd, Australia) were coated with 100μl Protein A (2.5 μg) suspended in PBS and incubated overnight at 4°C. Wells were washed using wash buffer (Tris gelatine-0.05% Tween 20, pH 7.4) and blocked with blocking buffer (Tris/gelatin/BSA, pH 7.4) at 37°C for 2 h followed by washing of the wells thrice and adding 100μl of 2-fold serial dilutions of test and negative control sera in triplicates. Plates were then incubated at 37°C for 2 h followed by re-washing of the wells using wash buffer. An aliquot of 100μl of 1: 10,000 dilution of alkaline phosphatase labelled goat anti-mouse IgA, IgM, IgG, IgG_1_, IgG_2a_ or IgG_2b_ were added to the wells and incubated at 4°C overnight. After washing for three times, substrate was added and the plates were read at 405 nm using ELISA plate reader (Model 550, BioRad). Specific absorbance versus serum dilutions were plotted to calculate antibody titres [41].

### Estimation of interferon gamma (IFN-γ) in splenocyte supernatants

Estimation of IFN-γ, as an indicator of cell mediated immunity (CMI) was carried out for the vaccinated and control groups of mice using ab46081 IFN-γ Mouse ELISA Kit (Abcam^®^) following manufacturer’s instructions. Collection of splenocyte supernatants and method for the stimulation of splenocytes cultured *in vitro* was carried out as described elsewhere [41].

### Determination of protective potential of Protein A using mouse mastitis model

A strong biofilm former *S*. *aureus* 51 with capsular polysaccharide type 8 and carrying both *ica*A and *ica*D loci was chosen [16] to be used in the challenge experiment. This strain was selected from the collection of 154 bovine mastitis-associated *S*. *aureus* strains obtained from Victoria and Queensland, Australia.

#### Preparation of bacterial inocula for challenge

*S*. *aureus* 51 was grown on Mueller-Hinton (MH) agar plates at 37°C for 18h as described previously [16] and the challenge suspension in isotonic saline adjusted to a final viable bacterial count of 4 x 10^11^ ml^-1^ as described previously [42].

#### Challenge experiment

Of the total 48 pregnant immunised mice, 24 mice (6 mice from each group, Table 1), representing s/c test versus s/c control, and i/mam test versus i/mam control groups, were euthanized on 28^th^ day for determining the immunogenicity of Protein A. The remaining 24 vaccinated mice, already in lactation, were challenged on days 5–15 of lactation. These mice were also divided into four groups of 6 mice each viz., s/c versus i/mam vaccination test groups and s/c versus i/mam control group (Table 1). Both L5 and R5 mammary glands of the lactating mice from each treatment group including the controls were injected with 50 μl (CFU 2x10^10^) [42] of *S*. *aureus* 51. Pups were removed from the lactating mice approximately 1h prior to infection of the mammary glands and euthanized post-anaesthesia, using 100 mg ketamine kg^−1^ and 10 mg xylazine kg^−1^, administered by the intraperitoneal (i/p) route which was followed by cervical dislocation. Mice were administered one dose of Buprenorphine hydrochloride (0.05–0.1 mg kg^-1^) subcutaneously prior to i/mam infection to offer anticipated pain relief for up to 12 h post-bacterial infection. Mice were then anaesthetized as described above and challenged via intra-mammary route using blunt 31G needles. Mice were observed at 6 hourly intervals in the first two days and then twice daily for morbidity and mortality until 5 days post-challenge. Percent protection against mouse mastitis was recorded and surviving mice euthanized after collecting blood samples using cardiac puncture, and collecting spleens and mammary glands. Cervical dislocation was performed to ensure death of the animals prior to disposal.

#### Clinical observations

The mammary glands of mice were observed for clinical signs of mastitis including redness, swelling and discolouration of mammary gland and extrusion of exudate with or without squeezing of the mammary gland. Monitoring of mice for morbidity and mortality was carried out at 6 hourly intervals up to 48h. The level of clinical signs was graded as 0 (no macroscopic changes), + (low) grade, ++ (medium grade) and +++ (severe grade) based on the observed clinical features including redness, swelling, and discolouration of mammary gland, exudate, morbidity and mortality.

#### Determination of bacterial loads of mammary glands

The bacterial loads of mammary glands post-challenge with *S*. *aureus* were determined as described previously [16]. Briefly, the L5 mammary glands from control and test mice were collected and subjected to bacterial load study. L5 mammary glands from both control and test mice were individually ground in sterile Griffith’s tubes containing 2 ml of sterile normal saline. Serial tenfold dilution from the homogenates from the mammary glands were inoculated on Baird Parker (BP) agar plates (Pathwest, Laboratory Medicine, WA) by the spread plate method and incubated at 37°C for 48h, followed by determination of colony counts of *S*. *aureus* per mammary gland.

#### Histopathological analysis

The method used for histopathological analysis of mammary glands post-challenge with *S*. *aureus* were determined as described previously [16]. Briefly, R5 mammary glands were collected from control and test mice were collected for histopathological examination. The glands were fixed in 10% neutral buffered formalin for 24 h, processed on an automatic tissue processor and embedded in paraffin wax. Tissue sections were cut at 4 μm thickness at three levels and stained by the Haematoxylin and Eosin stain [43]. An additional section was stained for bacteria using Gram Twort Method [44,45]. Grading of histological changes observed in mammary glands is as follows:

**Level 0:** No reaction.
**Level 1**: Organisms identified with minimal inflammatory response in mammary tissue.
**Level 2:** Moderate inflammation in peri-mammary and intramammary tissue with intraluminal organisms observed.
**Level 3:** Marked inflammatory cell infiltration into mammary tissue in the presence of organisms with evidence of tissue degeneration including necrosis.

### Statistical analysis

To compare the antigen specific antibody levels, IFN-γ levels and log_10_ average number of bacteria (CFU) recovered from mammary glands between groups of mice immunized with the Protein A vaccine, student’s *t-*test was performed. Statistical significance was set at p < 0.05.

## Results

### Detection of antigen-specific antibody in sera samples of mice vaccinated with Protein A

**Fig 1:**
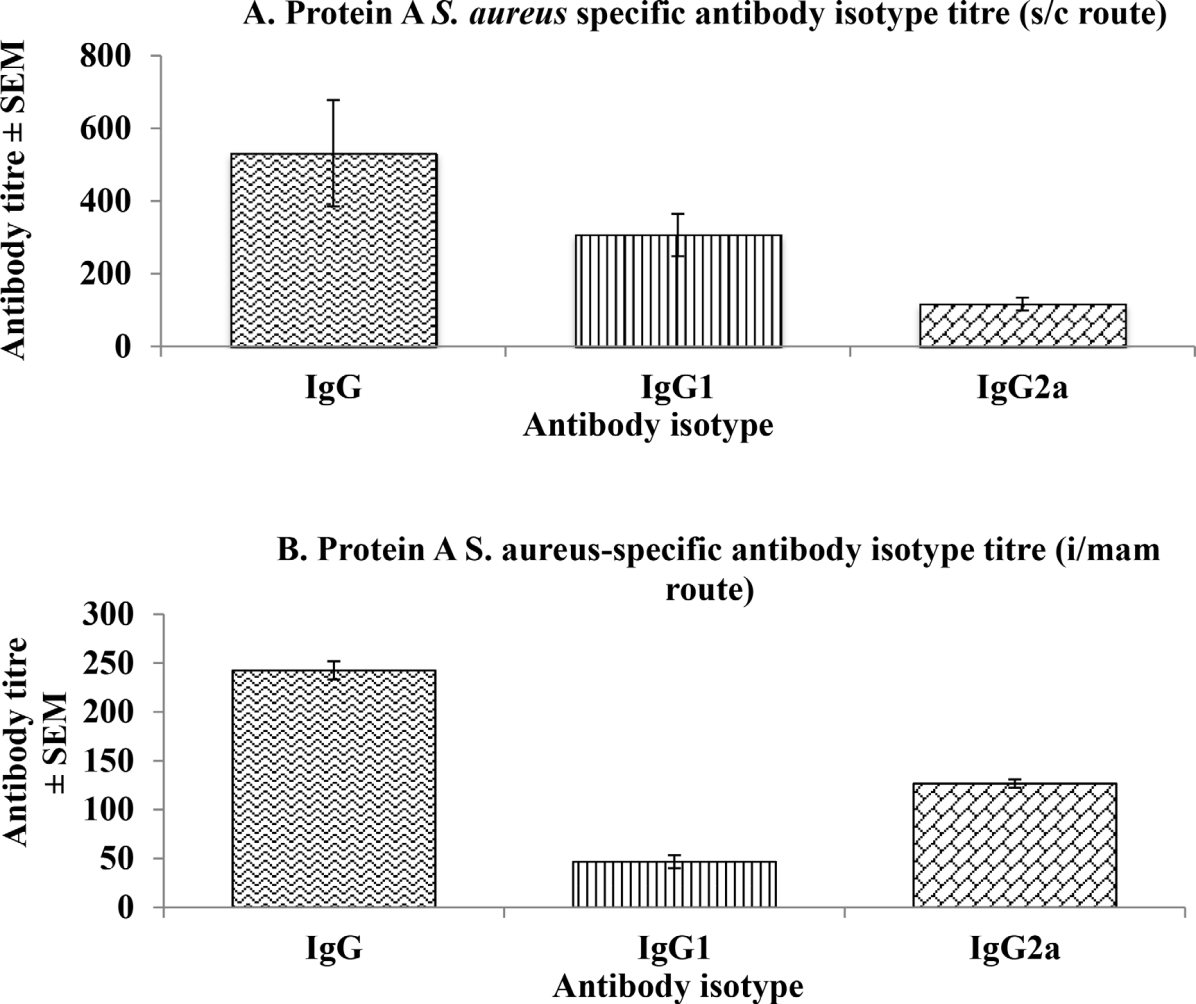
Antigen-specific antibody isotypes of mice vaccinated with Protein A by s/c or i/mam routes.

The titres of IgG, IgG_1_ and IgG_2a_ in mice vaccinated by s/c route were 531.67 ±146.21, 306.67±58.35 and 116.67±17.45, respectively (Fig 1A) and 242.5±9.29, 46.67±6.67 and 126.67±4.22, respectively in mice vaccinated by i/mam route (Fig 1B). The titres of serum antibody isotypes (IgG, IgG_1_) in mice immunized by i/mam route were significantly lower (p<0.05) than the mice immunized by s/c route except for IgG_2a._

### IFN-γ levels in splenocyte supernatants

**Fig 2:**
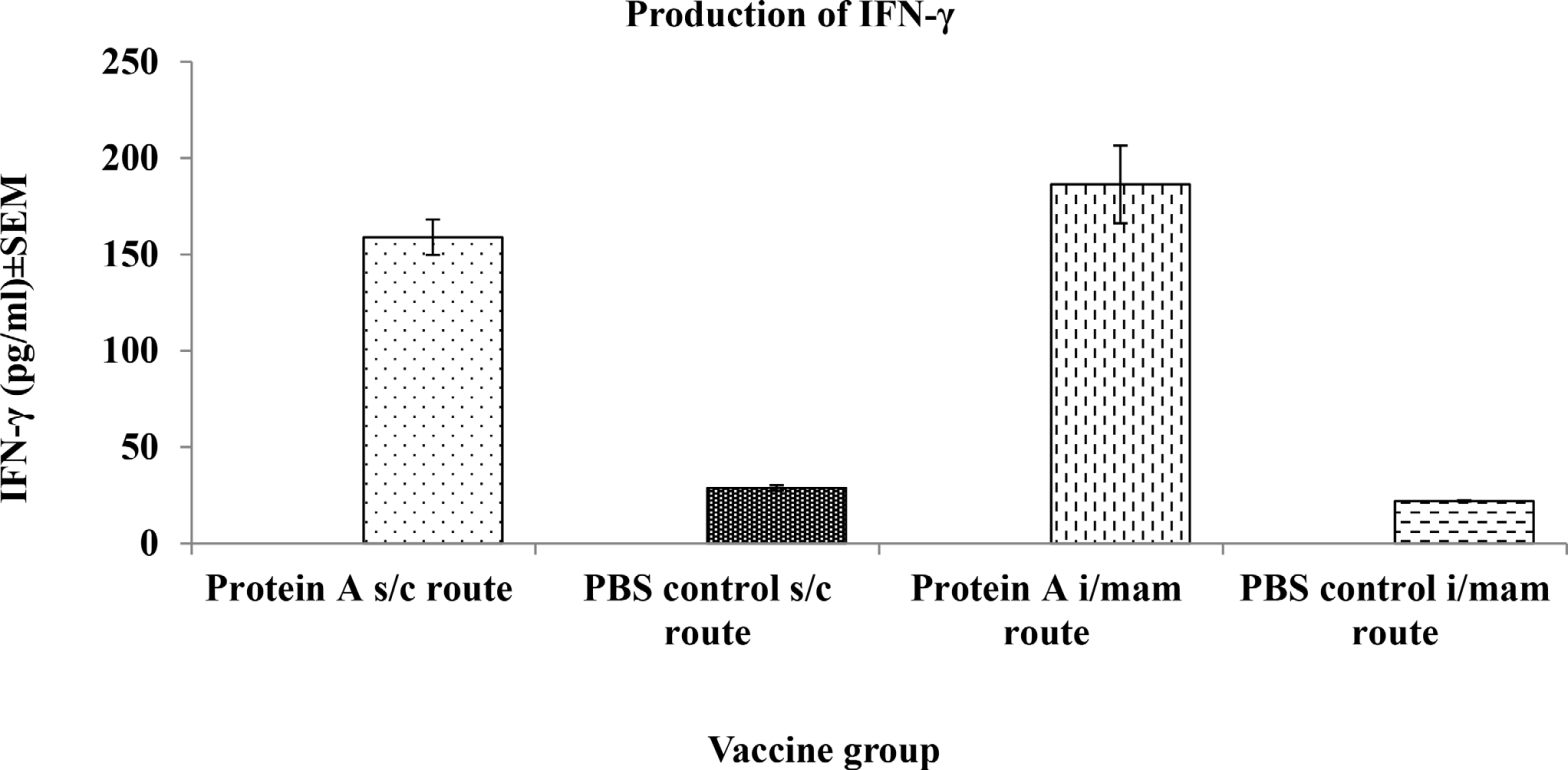
IFN-γ production by splenocytes of mice immunised with Protein A and PBS by s/c and i/mam routes following stimulation with Protein A.

Splenocytes of mice vaccinated with Protein A by both s/c and i/mam routes produced low levels of IFN-γ when stimulated with Protein A. However, splenocytes stimulated with ConA produced a high level of IFN-γ (5700 pg/ml) as expected. In the s/c and i/mam vaccination groups, IFN-γ levels produced by stimulation of splenocytes with Protein A 159±9.18 pg/ml and 186.34±20.09 pg/ml, respectively (Fig 2), which were not significantly different from each other.

### Clinical observation of mammary glands of challenged mice

The control group showed signs of mastitis in the inoculated glands. All the test mice showed varying degrees of changes in the gross clinical appearance of mammary glands (Table 2). Upon challenge with the biofilm forming *S*. *aureus* strain, there was no mortality of mice up to 5 days post-challenge. However, varied levels of clinical symptoms were observed. There was no difference between the levels of clinical symptoms observed in mice infected with PBS versus those vaccinated with Protein A by the s/c route. However, mice vaccinated with Protein A by i/mam route showed medium level of clinical symptoms up to day 3 post-challenge. On day 4 onwards there was no difference between the levels of clinical severity among all the groups of mice.

**Table 2:** Clinical signs observed in different groups of mice immunized with Protein A (Observations up to 5 days post challenge).

Level of clinical signs observed in test mice injected with Protein A					
Vaccination	Day1	Day2	Day3	Day4	Day5
ProteinA (s/c route)	++	+++	+++	+++	+++
ProteinA (i/mam route)	++	++	++	+++	+++
PBS control (s/c route)	++	+++	+++	+++	+++
PBS control (i/mam route)	++	+++	+++	+++	+++

Clinical features include redness, swelling, and discolouration of mammary gland, exudate, morbidity and mortality. Grade scores compare observed features to the most severe changes: 0—no macroscopic changes, + low grade, ++ medium grade, +++ severe grade.

### Bacterial loads in the mammary glands of challenged mice

There was significant difference (p<0.05) between the average bacterial load of mammary gland of mice vaccinated with Protein A by i/mam route was 7.33±0.29 CFU versus 8.18±0.14 CFU by the s/c route (Table 3). However, the bacterial loads of the mammary glands of control mice were significantly higher (p<0.05) than those of the vaccinated mice regardless of the route of immunization.

**Table 3:** Detection of bacterial load and histopathological changes in the mammary glands of mice immunized with Protein A.

Group	Vaccine group	Total number of mammary glands investigated	Log_10_ average number of bacteria (CFU) recovered from mammary glands ± SE	Grades for histopathological changes					
				M*1	M2	M3	M4	M5	M6
1	Protein A s/c route	6	8.18±0.14	3	3	3	3	3	2
2	Protein A i/mam route	6	7.33±0.29	3	3	3	3	2	3
3	Control s/c route	6	9.04±0.06	3	3	3	3	3	3
4	Control i/mam route	6	9.05±0.04	3	3	3	3	3	3

*M = Mammary gland

### Histopathological observation of mammary glands of challenged mice

**Fig 3:**
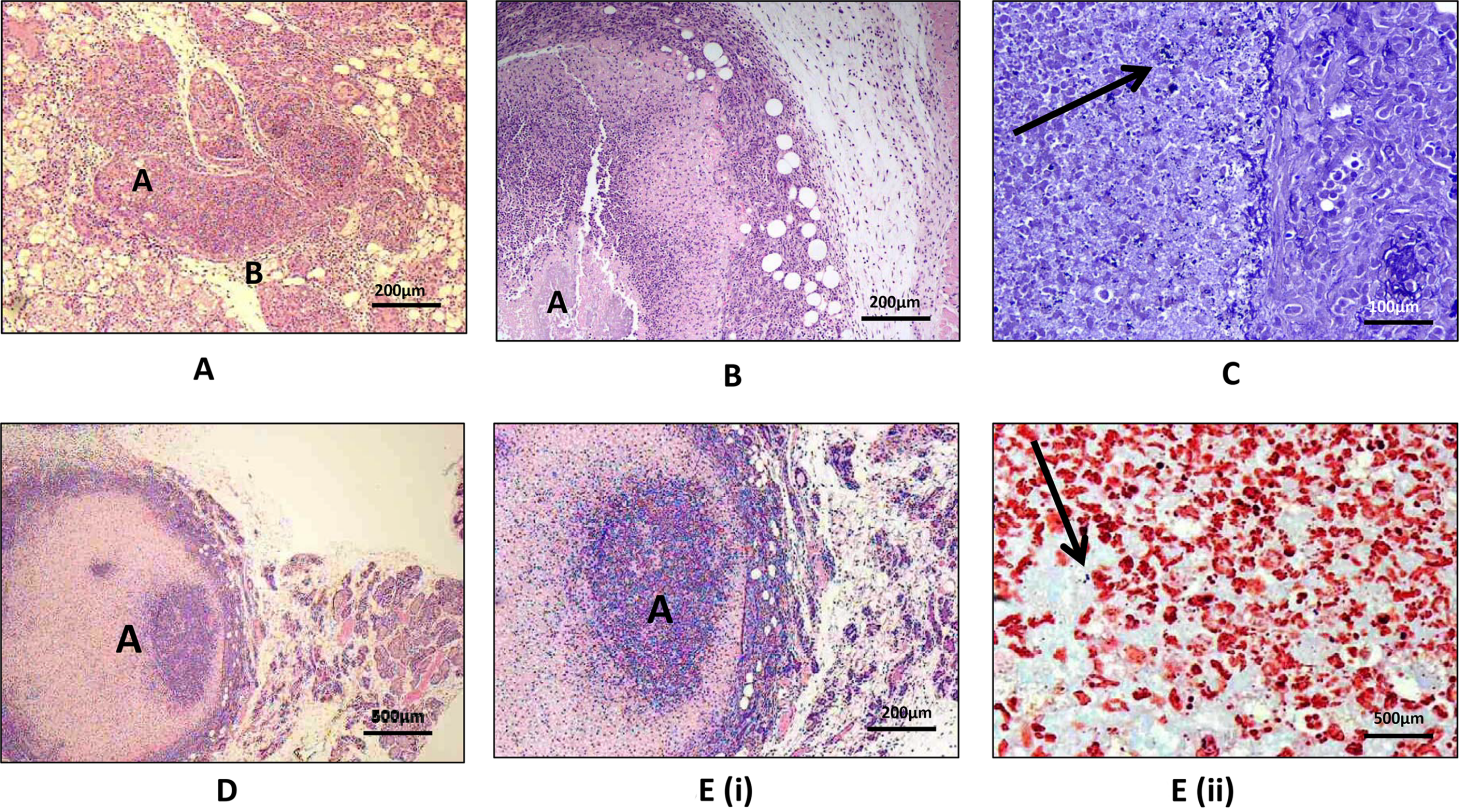
Fig 3A; Mammary tissue specimen from lactating mouse immunized with Protein A vaccine (s/c route) and challenged using strong biofilm forming *S*. *aureus* 51. Marked inflammatory cell infiltration within intralobular (A) and perilobular tissue (B). Level 3 category inflammation. H&E x 100, bar, 200 μm; Fig 3B; Mammary tissue specimen from lactating mouse immunized by Protein A vaccine (i/mam route) and challenged using strong biofilm forming *S*. *aureus* 51. Abscess in mammary tissue (A). Level 3 category inflammation. H&E x 100, bar, 200 μm; Fig 3C; Mammary tissue specimen from lactating mouse immunized by Protein A vaccine (i/mam route) and challenged using strong biofilm forming *S*. *aureus* 51. Gram positive bacteria and associated inflammatory cell infiltrate (Arrow). Gram Twort x 400, bar, 100 μm; Fig 3D; Mammary tissue specimen from lactating mouse injected with PBS and challenged using strong biofilm forming *S*. *aureus* 51. Abscess in mammary tissue with tissue necrosis (A). Level 3 category inflammation. H&E x 40, bar, 500 μm; Fig 3E; Mammary tissue specimen from lactating mouse infected with PBS and challenged using strong biofilm forming *S*. *aureus* 51(from Fig 3D); Fig 3E(i) Inflammatory abscess shows inflammatory exudate in fibrinous capsule (A). Level 3 category inflammation. H&E x 100, bar, 200 μm; Fig 3E(ii) Gram positive bacteria (Arrow) in inflammatory material Gram Twort x1000, bar, 500 μm.

Histopathological grade observed in the mammary tissue of control versus vaccinated groups of mice are shown in Table 3 and Fig 3A–3E. No significant differences were detected in histopathological grades of mammary tissue lesions between Protein A-vaccinated and control groups of mice.

## Discussion and Conclusions

Protein A is a single polypeptide chain of molecular weight 42,000 and contains little or no carbohydrate [46]. This molecule is one of the MSCRAMMs produced by *S*. *aureus*, has been reported to assist *S*. *aureus* in evading adaptive immune responses due to its’ antiphagocytic properties owing to its’ ability to bind the Fcγ domain of immunoglobulin G (IgG) and cross-linking of the Fab domain of VH3-type B cell receptors (IgM) [10,47]. Falugi and co-workers determined the immunogenicity and protective potential of non-Fc and non-Fab binding domain of the spA molecule and reported a significant reduction in abscess formation in kidneys of mice challenged with *S*. *aureus* by the intravenous route [38]. Unfortunately, primary immunisation was carried out using Freund’s complete adjuvant, disallowed in any vaccine formulation against human or animal infectious diseases. It would be important to know whether the same pattern of immunogenicity and protective potential will be observed if immunization of mice was carried out with antigens emulsified with the adjuvants permitted for use in humans or animals such as aluminium-based adjuvants or ASO4, combination of aluminium hydroxide and monophosphoryl A.

Considering the potential role of Protein A in the pathogenesis of staphylococcal infections [10,48], the present study was undertaken to test the immunogenicity and protective potential of Protein A in its’ native state against mouse mastitis caused by *S*. *aureus*. The study has demonstrated that mice vaccinated by subcutaneous or intra-mammary routes produced IgG, IgG1 (humoral immune response) and IgG2a, the latter being significantly greater (p<0.05) in mice vaccinated by the i/mam than the s/c route. A more likely explanation is the choice of alum as the adjuvant, which has been reported to preferentially promote Th2 cells, associated with IgG1 production and considered to be important for protection against infection against extracellular pathogens. These adjuvants are less effective at promoting Th1 cells reported to be associated with IgG2a production [49,50]. Clearly, this was not the case in Protein A-vaccinated mice since the level of IFN-γ was not significantly different regardless of the route of immunization. Therefore, the inability of Protein A to protect lactating mice against mouse mastitis may have been due to the inability of this molecule to promote induction of CMI.

Interestingly, the bacterial loads of the mammary glands of all the four groups of mice, including s/c test, s/c control i/mam test, and i/mam control, respectively, were high except in mice vaccinated by the i/mam route, which had significantly lower loads (p<0.05) than the s/c group and sham-vaccinated controls. In our study, histopathological analysis of the tissue sections did not demonstrate any differences between the mice immunized with Protein A by the subcutaneous or intra-mammary routes and the unvaccinated control group of mice. All groups of mice including sham-vaccinated controls showed almost identical mammary tissue damage indicating the lack of usefulness of Protein A as a vaccine candidate for inclusion in the formulation of conjugate vaccines against bovine mastitis caused by *S*. *aureus*. Our findings are indirectly supported by those of Greenberg and co-workers [24] who reported the inability of Protein A-specific antisera to offer protection against bacteraemia, metastatic infection of lungs and liver of infant rats following systemic challenge with *S*. *aureus*. In contrast, Pankey and co-workers reported the protective potential of *S*. *aureus* bacterin and Protein A against experimental mastitis in cows. They reported spontaneous cure rate from experimental mastitis in cows induced by intra-mammary infection with *S*. *aureus* strain Mexi, as 83% and 73%, respectively [51]. Cows were immunised with *S*. *aureus* bacterin and Protein A emulsified in Freund’s complete adjuvant (FCA), followed by 2 booster vaccinations with the bacterin emulsified in Freund’s incomplete adjuvant. Unfortunately, no analyses of the immune responses that were induced post-vaccination of cows with either the *S*. *aureus* bacterin or Protein A were reported.

Given the inability of Protein A to offer protection against mastitis in the mouse mastitis model, it may be reasonable to conclude that Protein A in its’ native state may not be a priority vaccine candidate for inclusion in a vaccine formulation, with or without conjugation with capsular polysaccharides and/or PNAG, for the prevention of bovine mastitis. However, it may be interesting to determine the immunogenicity and protective potential of Protein A in its’ native state in a novel adjuvant capable of promoting the induction of CMI and the non-Fc (γ or μ) or Fab binding variant of Protein A (SpA_kkaa_), emulsified in a suitable adjuvant approved for use in humans or animals, against bovine mastitis, using the mouse mastitis model.
